# Supplementary material for: On the excitation and radiative decay rates of plasmonic nanoantennas
Source: Nanophotonics. 2022 Mar 28;11(10):2271–81. doi: 10.1515/nanoph-2022-0015 (PMC11635946; doi:10.1515/nanoph-2022-0015)
Supplement: Supplementary file 1 — Supplementary Material Details [file j_nanoph-2022-0015_suppl.pdf]

Supplementary Information: On the Excitation and Radiative Decay Rates of Plasmonic Nanoantennas

Kalun Bedingfield and Angela Demetriadou*

School of Physics and Astronomy, University of Birmingham, Edgbaston, Birmingham, B15 2TT, United Kingdom

E-mail: a.demetriadou@bham.ac.uk

S1 Non-Radiative Decay Channels of Nanoantennas

Here we introduce the non-radiative decay rate (γ_{nrad}) to the comparison of the γ_{exc} and γ_{rad} seen in Figure 1 of the main manuscript. In all cases we observe a prominent peak at ∼530nm due to the collective response of the higher order modes' dark nature, as shown in Figure S1. For the isolated NP in Figure S1(a), the non-radiative response is orders of magnitude larger than both the γ_{exc} and γ_{rad}. For the quasistatic dimer in Figure S1(b), the hybridisation within the cavity leads to the red-shifting of the l =1 mode, as well as it's enhancement. Despite this enhancement, the γ_{nrad} of the $l = 1$ mode is still an order or magnitude larger than the γ_{exc} and γ_{rad}—although this has reduced significantly compared to the isolated NP. This behaviour is also observed for the NPoM system in Figure S1(c), with a proportional increasing in the γ_{rad} even though the overall intensity of the γ_{nrad} has reduced slightly. Interestingly, there is minimal enhancement of the higher order dark modes—even for such a small cavity—due to the spatial distributions of the higher order modes as well

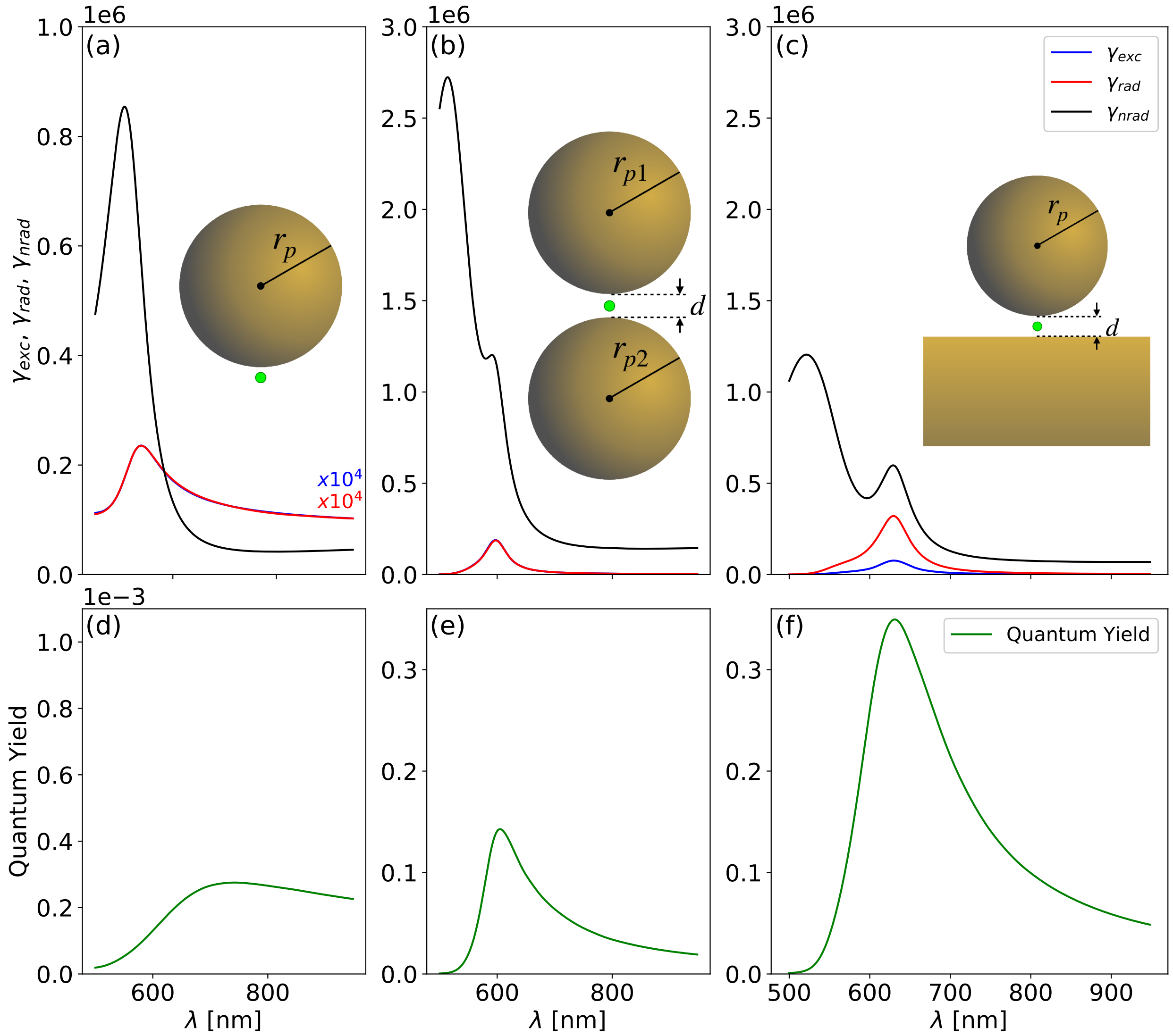

Figure S1: Numerical comparison of the γ_{exc} (blue) and γ_{rad} (red) with the γ_{nrad} (black) in different nanoplasmonic systems. The green dot indicates the position of the QE when determining the γ_{rad} and γ_{nrad}, and the location where the fields are measured when calculating the γ_{exc}. (a) Isolated $2r_p = 60$nm gold NP. (b) Dimer of $2r_{p1} = 2r_{p2} = 60$nm gold NPs with a $d = 1$nm separation. (c) NPoM geometry for a $2r_p = 60$nm gold NP and a $d = 1$nm separation. Figures (d)-(f) compare the corresponding quantum yield for the systems in (a)-(c).

as their confinement closer to the surface of the NP. As such, it becomes clear why the selective enhancement regions of such nanoantennas is invaluable for its ability to not only increase the radiative properties by orders of magnitudes, but to do so relative to the γ_{nrad}. Figures S1(d)-(f) show the corresponding quantum yield for these three systems, comparing the γ_{rad} to the total decay rate of the system. This highlights the radiative efficiency of these systems compared to the losses attributed to the higher order dark modes.

Similarly to the NPoM system, the comparison of the γ_{nrad} to the in- and out-coupling for an asymmetric dimer system is considered in Figure S2, and shows the same trend.

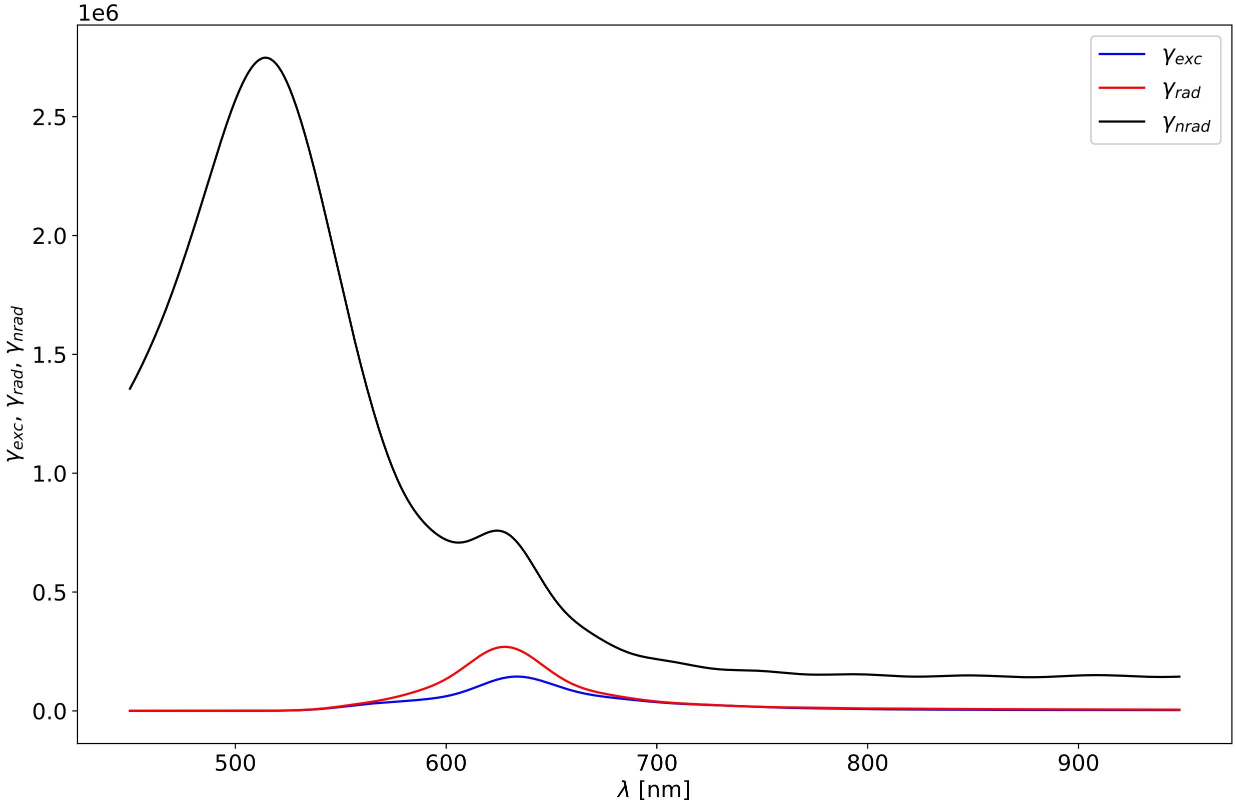

Figure S2: Numerical comparison of the γ_{exc} (blue) and γ_{rad} (red) with the γ_{nrad} (black) for an asymmetric dimer system consisting of a $2r_{p,1} = 60$nm gold NP sat $d = 1$nm above a $2r_{p,2} = 1\mu$m gold NP.

S2 Multipole expansion for the electromagnetic fields of an Isolated Nanoparticle

The electromagnetic fields emerging from a dipole source exciting an isolated spherical NP can be obtained by solving the vector Helmholtz equation for $\mathbf{E}$ and $\mathbf{H}$. However, it is convenient to solve the problem in a spherical coordinate system, since the NP is spherical, and also convenient to reduce the Helmholtz equation to a scalar equation:

$$\left[\nabla^2 + k^2\right]\psi = 0 \tag{S1}$$

The solutions to the above homogeneous scalar Helmholtz equation lead to the scalar wavefunctions:

$$\psi^{e,o}_{l,m}(\mathbf{r}) = z_l(kr)P^m_l(\cos\theta)\begin{cases} \cos m\phi \\ \sin m\phi \end{cases} \tag{S2}$$

where $z_l(kr)$ is a general spherical Bessel function and here we use either the first $(j_l(kr))$ or the third kind (Hankel, $h^{(1)}_l(kr)$) functions. The scalar wavefunctions can then be used to build the spherical vector harmonics as:

$$\begin{aligned} \mathbf{M}^{e,o}_{l,m} &= \nabla \times \left(\mathbf{r}\psi^{e,o}_{l,m}\right) \\ \mathbf{N}^{e,o}_{l,m} &= \frac{1}{k}\nabla \times \mathbf{M}^{e,o}_{l,m} \end{aligned} \tag{S3}$$

which are the solutions to the homogeneous vector Helmholtz equation and given by:

$$\mathbf{M}_{l,m}^{e,o}(k\mathbf{r}) = \begin{pmatrix} 0 \\ \frac{m}{\sin\theta} z_l(kr) P_l^m(\cos\theta) \begin{cases} -\sin m\phi \\ \cos m\phi \end{cases} \\ -z_l(kr)\frac{\partial P_l^m(\cos\theta)}{\partial\theta} \begin{cases} \cos m\phi \\ \sin m\phi \end{cases} \end{pmatrix}$$

$$\mathbf{N}_{l,m}^{e,o}(k\mathbf{r}) = \begin{pmatrix} l(l+1) z_l(kr) p_l^m(\cos\theta) \begin{cases} \cos m\phi \\ \sin m\phi \end{cases} \\ \left[kr z_l(kr)\right]' P \frac{\partial P_l^m(\cos\theta)}{\partial\theta} \begin{cases} \cos m\phi \\ \sin m\phi \end{cases} \\ \frac{m}{\sin\theta}\left[kr z_l(kr)\right]' P_l^m(\cos\theta) \begin{cases} -\sin m\phi \\ \cos m\phi \end{cases} \end{pmatrix} \quad \text{(S4)}$$

Without loss of generality, the incident Green's dyadic tensor for a dipole source placed in free space at $\mathbf{r}'$ is given by:

$$\overleftrightarrow{G}_{inc}(\mathbf{r},\mathbf{r}') = \sum_L C_{l,m} \begin{cases} \mathbf{M}_{l,m}^{e,o\ (1)}(k\mathbf{r}') \otimes \mathbf{M}_{l,m}^{e,o\ (3)}(k\mathbf{r}) + \mathbf{N}_{l,m}^{e,o\ (1)}(k\mathbf{r}') \otimes \mathbf{N}_{l,m}^{e,o\ (3)}(k\mathbf{r}) & r > r' \\ \mathbf{M}_{l,m}^{e,o\ (3)}(k\mathbf{r}') \otimes \mathbf{M}_{l,m}^{e,o\ (1)}(k\mathbf{r}) + \mathbf{N}_{l,m}^{e,o\ (3)}(k\mathbf{r}') \otimes \mathbf{N}_{l,m}^{e,o\ (1)}(k\mathbf{r}) & r < r' \end{cases} \quad \text{(S5)}$$

where we used the abbreviated notation $\sum_L = \sum_{l,m}\sum_{e,o}$ and $C_{l,m} = \frac{ik}{4\pi}(2-\delta_0)\frac{2l+1}{l(l+1)}\frac{(l-m)!}{(l+m)!}$ with $\delta_0 = \begin{cases} 1, & m = 0 \\ 0, & m \neq 0 \end{cases}$. The superscripts $^{(1)}$ and $^{(3)}$ refer to the use of spherical Bessel functions of the first ($j_l(kr)$) and third (Hankel, $h_l^{(1)}(kr)$) kinds respectively for the general Bessel function $z_l(kr)$ in $\psi_{l,m}^{e,o}$.

It follows that the scattered and internal Green's dyadic tensor are given by:

$$\begin{aligned}
\overleftrightarrow{G}_{scat}(\mathbf{r},\mathbf{r}') &= -\sum_L C_{l,m}\left[b_l \mathbf{M}^{e,o\;(3)}_{l,m}(k\mathbf{r}') \otimes \mathbf{M}^{e,o\;(3)}_{l,m}(k\mathbf{r}) + a_l \mathbf{N}^{e,o\;(3)}_{l,m}(k\mathbf{r}') \otimes \mathbf{N}^{e,o\;(3)}_{l,m}(k\mathbf{r})\right] \quad \text{(S6)}\\
\overleftrightarrow{G}_{int}(\mathbf{r},\mathbf{r}') &= \sum_L C_{l,m}\left[c_l \mathbf{M}^{e,o\;(3)}_{l,m}(k\mathbf{r}') \otimes \mathbf{M}^{e,o\;(1)}_{l,m}(Nk\mathbf{r}) + d_l \mathbf{N}^{e,o\;(3)}_{l,m}(k\mathbf{r}') \otimes \mathbf{N}^{e,o\;(1)}_{l,m}(Nk\mathbf{r})\right]
\end{aligned}$$

where N is the refractive index of the materials forming the NP and a_l, b_l, c_l, d_l is a set of l-dependent coefficients. These coefficients can be determined from the boundary conditions at the surface of the NP ($r = r_p$), where the tangential fields needs to be continuous:

$$(\mathbf{E}_{inc} + \mathbf{E}_{scat} - \mathbf{E}_{int})\,|_{r=r_p} \times \hat{\mathbf{e}}_{\mathbf{r}} = 0 \qquad \text{(S7)}$$

$$(\mathbf{H}_{inc} + \mathbf{H}_{scat} - \mathbf{H}_{int})\,|_{r=r_p} \times \hat{\mathbf{e}}_{\mathbf{r}} = 0 \qquad \text{(S8)}$$

and are given by[1]:

$$\begin{aligned}
a_l &= \frac{N^2 j_l(Nkr_p)[kr_p j_l(kr_p)]' - j_l(kr_p)[Nkr_p j_l(Nkr_p)]'}{N^2 j_l(Nkr_p)[kr_p h_l^{(1)}(kr_p)]' - h_l^{(1)}(kr_p)[Nkr_p j_l(Nkr_p)]'} && \text{(S9)}\\
b_l &= \frac{j_l(Nkr_p)[kr_p j_l(kr_p)]' - j_l(kr_p)[Nkr_p j_l(Nkr_p)]'}{j_l(Nkr_p)[kr_p h_l^{(1)}(kr_p)]' - h_l^{(1)}(kr_p)[Nkr_p j_l(Nkr_p)]'} && \text{(S10)}\\
c_l &= \frac{j_l(kr_p)[kr_p h_l^{(1)}(kr_p)]' - h_l^{(1)}(kr_p)[kr_p j_l(kr_p)]'}{j_l(Nkr_p)[kr_p h_l^{(1)}(kr_p)]' - h_l^{(1)}(kr_p)[Nkr_p j_l(Nkr_p)]'} && \text{(S11)}\\
d_l &= \frac{N j_l(kr_p)[kr_p h_l^{(1)}(kr_p)]' - N h_l^{(1)}(kr_p)[kr_p j_l(kr_p)]'}{N^2 j_l(Nkr_p)[kr_p h_l^{(1)}(kr_p)]' - h_l^{(1)}(kr_p)[Nkr_p j_l(Nkr_p)]'}\,. && \text{(S12)}
\end{aligned}$$

The E-fields are then obtained by $\mathbf{E} = \omega^2\mu_0 \overleftrightarrow{G}(\mathbf{r},\mathbf{r}')\cdot\mathbf{p}(\mathbf{r}')$ where $\mathbf{p}(\mathbf{r}') = \mathbf{p}_0\delta(\mathbf{r}-\mathbf{r}')$ is the dipole moment of the dipole source at position $\mathbf{r}'$.

S3 From a dipole source to a plane wave incidence

From Section S2, the Green's dyadic for a dipole source in free space is given by:

$$\overleftrightarrow{G}(\mathbf{r},\mathbf{r}') = \sum_L C_{l,m} \begin{cases} \mathbf{M}^{e,o}_{l,m}{}^{(1)}(k\mathbf{r}') \otimes \mathbf{M}^{e,o}_{l,m}{}^{(3)}(k\mathbf{r}) + \mathbf{N}^{e,o}_{l,m}{}^{(1)}(k\mathbf{r}') \otimes \mathbf{N}^{e,o}_{l,m}{}^{(3)}(k\mathbf{r}) & r > r' \\ \mathbf{M}^{e,o}_{l,m}{}^{(3)}(k\mathbf{r}') \otimes \mathbf{M}^{e,o}_{l,m}{}^{(1)}(k\mathbf{r}) + \mathbf{N}^{e,o}_{l,m}{}^{(3)}(k\mathbf{r}') \otimes \mathbf{N}^{e,o}_{l,m}{}^{(1)}(k\mathbf{r}) & r < r' \end{cases} \tag{S13}$$

where we used the abbreviated notation $\sum_L = \sum_{l,m} \sum_{e,o}$ and $C_{l,m} = \frac{ik}{4\pi}(2-\delta_0)\frac{2l+1}{l(l+1)}\frac{(l-m)!}{(l+m)!}$ with $\delta_0 = \begin{cases} 1, & m = 0 \\ 0, & m \neq 0 \end{cases}$. The superscripts $^{(1)}$ and $^{(3)}$ refer to the use of spherical Bessel functions of the first ($j_l(kr)$) and third (Hankel, $h_l^{(1)}(kr)$) kinds respectively for the general Bessel function $z_l(kr)$ in $\psi^{e,o}_{l,m}$. The electric field emitted by the dipole source is given by:

$$\mathbf{E} = \omega^2 \mu_0 \overleftrightarrow{G}(\mathbf{r},\mathbf{r}') \cdot \mathbf{p}(\mathbf{r}') \tag{S14}$$

where $\mathbf{p}(\mathbf{r}') = \mathbf{p}_0 \delta(\mathbf{r} - \mathbf{r}')$.

A plane wave can be viewed as a dipole source placed at a large distance from the object ($r' > r$), so the fields of a plane wave are given by:

$$\mathbf{E} = \omega^2 \mu_0 \sum_L C_{lm} \left[s^{e,o(3)}_{lm}(k\mathbf{r}') \mathbf{M}^{e,o}_{l,m}{}^{(1)}(k\mathbf{r}) + t^{e,o(3)}_{lm}(k\mathbf{r}') \mathbf{N}^{e,o}_{l,m}{}^{(1)}(k\mathbf{r}) \right] \tag{S15}$$

where $s^{e,o(3)}_{lm}(k\mathbf{r}') = \mathbf{M}^{e,o}_{l,m}{}^{(3)}(k\mathbf{r}') \cdot \mathbf{p}(\mathbf{r}')$ and $t^{e,o(3)}_{lm}(k\mathbf{r}') = \mathbf{N}^{e,o}_{l,m}{}^{(3)}(k\mathbf{r}') \cdot \mathbf{p}(\mathbf{r}')$. For a certain position of the dipole source, $s^{e,o(3)}_{lm}(k\mathbf{r}')$ and $t^{e,o(3)}_{lm}(k\mathbf{r}')$ are constants and always scalars.

If we place an x-polarized dipole source at $\mathbf{r}' \to \infty$, which means that it has coordinates: $(r' \to \infty, \theta' = \pi, \phi' = 0)$, then its dipole moment is given by:

$$\mathbf{p}(\mathbf{r}') = p_0 \hat{\mathbf{e}}_x = p_0 \left[\sin\theta' \cos\phi' \hat{\mathbf{e}}_r + \cos\theta' \cos\phi' \hat{\mathbf{e}}_\theta - \sin\phi' \hat{\mathbf{e}}_{phi} \right] \tag{S16}$$

which reduces to:

$$\mathbf{p}(\mathbf{r}') = p_0 \hat{\mathbf{e}}_x = -p_0 \hat{\mathbf{e}}_\theta \tag{S17}$$

Therefore the product of the dipole moment with the spherical harmonics (and considering $\phi' = 0$), leads to $s_{lm}^{e(3)}(k\mathbf{r}') = t_{lm}^{o(3)}(k\mathbf{r}') = 0$, and the only remaining coefficients are:

$$\begin{aligned} s_{lm}^{o(3)}(k\mathbf{r}') &= -p_0 h_l^{(1)}(kr') \frac{m}{\sin\theta'} P_l^m(\cos\theta') \\ t_{lm}^{e(3)}(k\mathbf{r}') &= -p_0 \frac{1}{kr'} [kr' h_l^{(1)}(kr')]' \frac{\partial P_l^m(\cos\theta')}{\partial\theta'} \end{aligned} \tag{S18}$$

where $[\cdot]'$ refers to the derivative with respect to the Hankel function's argument. The Hankel function, and its derivative, in the asymptotic limit where $kr' \gg l^2$ can be written as:

$$\begin{aligned} h_l^{(1)} &\sim \frac{(-i)^l e^{ikr'}}{ikr'} \\ \left[kr' h_l^{(1)}(kr')\right]' &\sim \frac{(-i)^l e^{ikr'}}{ikr'}(1 + ikr') \end{aligned} \tag{S19}$$

Finally, we need to obtain the values of the Associated Legendre Polynomials for $\theta' = \pi$. The Associated Legendre Polynomial takes non-zero values for $\theta' = \pi$, only when $m = 0$. However, this leads to the term $\frac{m}{\sin\theta'} P_l^m(\cos\theta')$ been undefined. Instead, we use an identity of $P_l^m(\cos\theta')$, which allows us to write this term as:

$$\frac{m}{\sin\theta'} P_l^m(\cos\theta') = -\frac{1}{2}\left[P_{l+1}^{m+1}(\cos\theta') + (l+m-1)(l+m) P_{l-1}^{m-1}(\cos\theta')\right] \tag{S20}$$

which for $\theta' = \pi$ only takes non-zero values for $m = \pm 1$. We choose the $m = 1$ solution and obtain:

$$\begin{aligned} \frac{m}{\sin\theta'} P_l^m(\cos\theta')\Big|_{\theta'=\pi} &= -\frac{1}{2}\left[P_{l+1}^2(-1) + l(l+1) P_{l-1}^0(-1)\right] \\ &= -\frac{1}{2} l(l+1)(-1)^{l-1} \end{aligned} \tag{S21}$$

where we used $P^2_{l+1}(-1) = 0$ and $P^0_{l-1}(-1) = (-1)^{l-1}$. Similarly for:

$$\left.\frac{\partial P_l^m(\cos\theta')}{\partial\theta'}\right|_{\theta'=\pi} = -\frac{1}{2}l(l+1)(-1)^l \tag{S22}$$

Therefore, the two coefficients are given by:

$$\begin{aligned} s_{lm}^{o(3)}(k\mathbf{r}') &= -p_0\frac{1}{2}l(l+1)\frac{i^l e^{ikr'}}{ikr'} \\ t_{lm}^{e(3)}(k\mathbf{r}') &= p_0\frac{1}{2}l(l+1)i\frac{i^l e^{ikr'}}{ikr'} \end{aligned} \tag{S23}$$

where we took $(\frac{1}{kr'} + i) \to i$. Substituting back into (S14), the E-fields generated by a dipole source placed at $(r' \to \infty, \theta' = \pi, \phi' = 0)$ are given by:

$$\mathbf{E}(\mathbf{r}) = -\frac{p_0 e^{ikr'}}{4\pi r'} i^l \frac{(2l+1)}{l(l+1)}\omega^2\mu_0 \left[\mathbf{M}^{o\ (1)}_{l,m}(k\mathbf{r}) - i\mathbf{N}^{e\ (1)}_{l,m}(k\mathbf{r})\right] \tag{S24}$$

which is in the same form as an x-polarized plane wave propagating along the z-axis.

S4 Radiative Decay Rate of an Isolated Nanoparticle System

The γ_{rad} is given by the energy crossing a putative sphere of radius $R = 1m$ that encloses the system in question. The rate at which energy passes through this sphere is given by[2]:

$$W_{total} = \int \mathbf{S}_{total}(\mathbf{r}) \cdot \hat{\mathbf{n}} dA \tag{S25}$$

where $\mathbf{S}_{total}(\mathbf{r})$ is the total Poynting vector at location $\mathbf{r}$, and $\hat{\mathbf{n}}$ is the unit vector normal to the surface of the putative sphere enclosed by dA. The total Poynting vector is given by:

$$\mathbf{S}_{total}(\mathbf{r}) = \frac{1}{2}\mathrm{Re}\left[\mathbf{E}_{total}(\mathbf{r}) \times \mathbf{H}^{*}_{total}(\mathbf{r})\right] \tag{S26}$$

The total fields are the summation of incident and scattered fields and when expanded leads to the total Poynting vector given by:

$$\mathbf{S}_{total}(\mathbf{r}) = \mathbf{S}_{inc}(\mathbf{r}) + \mathbf{S}_{scat}(\mathbf{r}) + \mathbf{S}_{ext}(\mathbf{r}) \tag{S27}$$

where $\mathbf{S}_{inc}$, $\mathbf{S}_{scat}$ and $\mathbf{S}_{ext}$ is the energy reaching the putative sphere directly from the source, the scattered fields from the nanoparticle and the energy extinct from the source respectively.

To obtain the radiative decay rate (γ_{rad}), we need to find the total energy crossing the putative sphere (W_{total}) and normalize it to the incident energy crossing the same sphere (W_{inc}):

$$\gamma_{rad} = \frac{W_{total}}{W_{inc}} = \frac{\int \mathbf{S}_{total}(\mathbf{r}) \cdot \hat{\mathbf{n}} dA}{\int \mathbf{S}_{inc}(\mathbf{r}) \cdot \hat{\mathbf{n}} dA} \tag{S28}$$

which leads to:

$$\gamma_{rad} = \frac{\sum_L (2-\delta_0)\, C_{l,m} \left[\left| s_{l,m}^{e,o\ (1)}(k\mathbf{r}') - b_l\ s_{l,m}^{e,o\ (3)}(k\mathbf{r}') \right|^2 + \left| t_{l,m}^{e,o\ (1)}(k\mathbf{r}') - a_l\ t_{l,m}^{e,o\ (3)}(k\mathbf{r}') \right|^2 \right]}{\sum_L (2-\delta_0)\, C_{l,m} \left[\left| s_{l,m}^{e,o\ (1)}(k\mathbf{r}') \right|^2 + \left| t_{l,m}^{e,o\ (1)}(k\mathbf{r}') \right|^2 \right]} \tag{S29}$$

where $s_{l,m}^{e,o\ (1)}(k\mathbf{r}') = \mathbf{M}_{l,m}^{e,o\ (1)}(k\mathbf{r}') \cdot \mathbf{p}(\mathbf{r}')$ and $t_{l,m}^{e,o\ (1)}(k\mathbf{r}') = \mathbf{N}_{l,m}^{e,o\ (1)}(k\mathbf{r}') \cdot \mathbf{p}(\mathbf{r}')$ are scalar coefficients that describe how efficiently a dipole source at $\mathbf{r}'$ couples energy into the free space modes, and $s_{l,m}^{e,o\ (3)}(k\mathbf{r}') = \mathbf{M}_{l,m}^{e,o\ (3)}(k\mathbf{r}') \cdot \mathbf{p}(\mathbf{r}')$ and $t_{l,m}^{e,o\ (3)}(k\mathbf{r}') = \mathbf{N}_{l,m}^{e,o\ (3)}(k\mathbf{r}') \cdot \mathbf{p}(\mathbf{r}')$ are also scalar coefficients describing how a dipole source at $\mathbf{r}'$ couples energy into the photonic modes of the system. The coefficients a_l and b_l are the two scattering Mie coefficients obtained in Section S2, and describe how efficiently the NP scatters energy.

S5 Excitation Rate Solver Comparison

Figure S3 compares the analytical γ_{exc} with the numerical values found from two different softwares: FDTD[3] and COMSOL[4]. This highlights the variance and sensitivity of the in- and out-coupling rates with meshing.

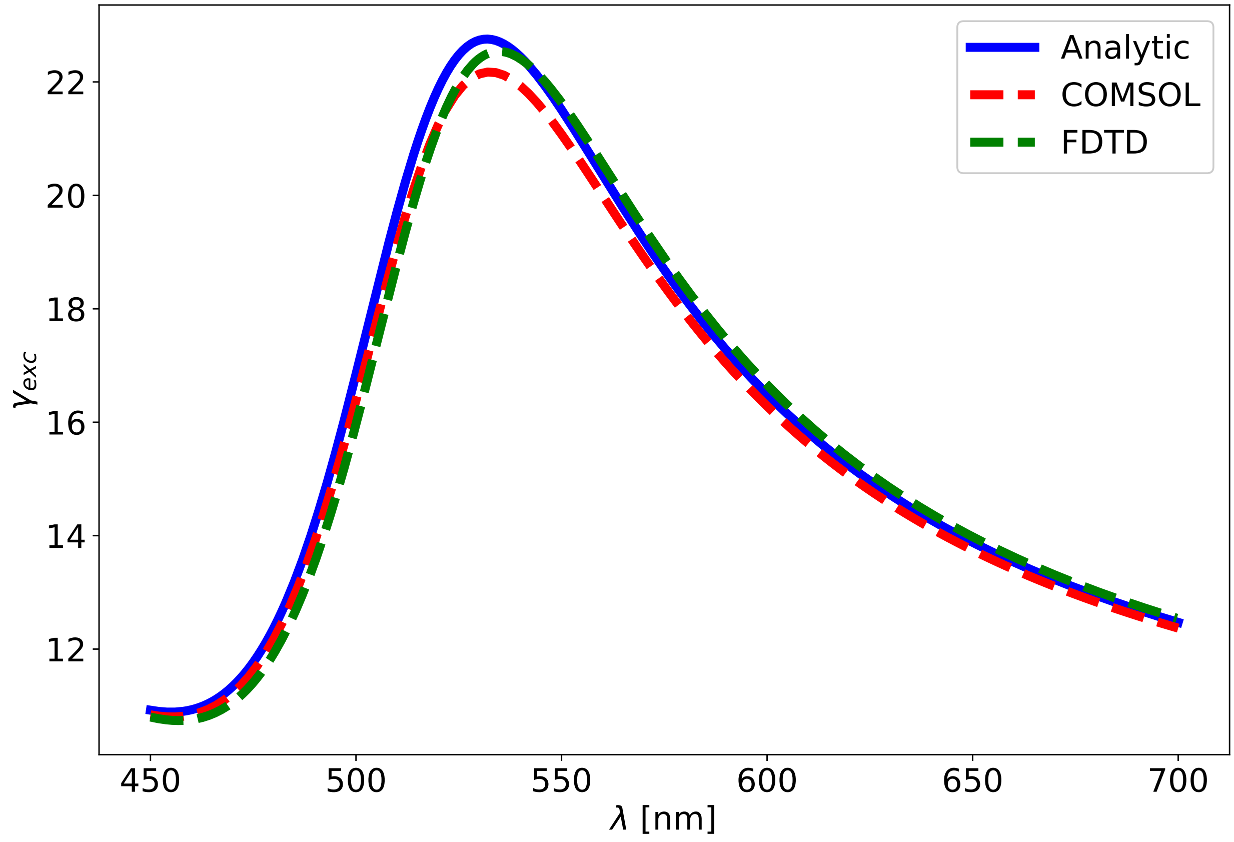

Figure S3: The γ_{exc} for an isolated spherical NP of diameter 60nm. The analytical multipolar decompositions (blue, full lines) is plotted together numerical calculations for comparison: COMSOL (red, dashed lines) and FDTD (green, dashed lines).

S6 Multipolar Decomposition of an Isolated Nanoparticle's Emission and Excitation

From the Green's function formalism, the γ_{exc} and γ_{rad} can be decomposed into their modal contributions. For a 1μm diameter isolated gold NP, the multipolar decomposition highlights the series of higher order modes that are efficiently excited—as shown in Figure S4.

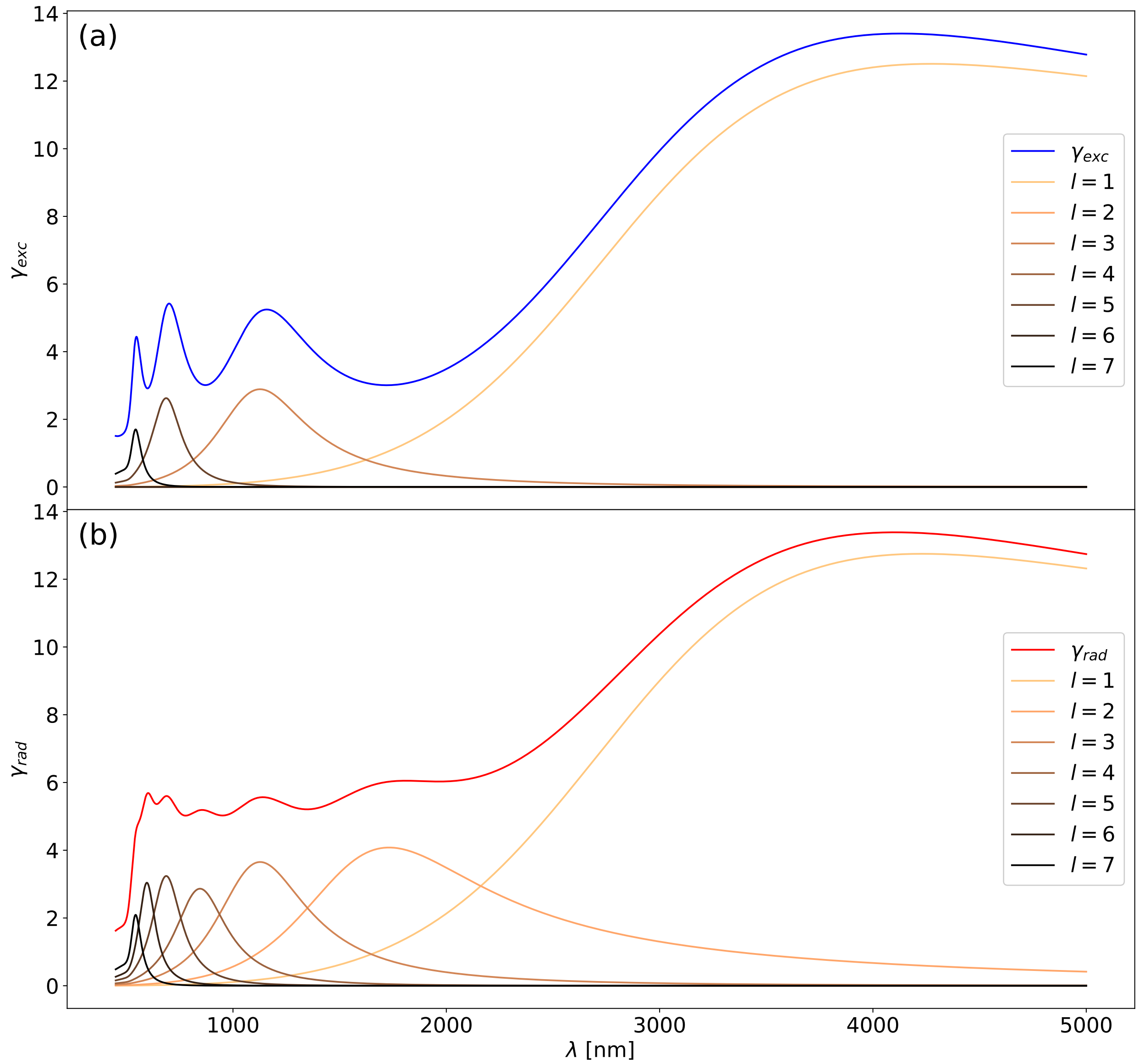

Figure S4: An analytical modal decomposition of the (a) γ_{exc} and (b) γ_{rad} for a 1μm diameter isolated gold NP, due to an x-polarised QE placed 0.5nm above the NP in the x-axis.

It is important to note the breadth of the tails of the Lorentzian poles, and the impact they will have in forming nanoantenna geometries that exhibit equal in- and out-coupling.

S7 Selective Nature of Plane Wave Excitations

A QE emits with wavevectors in all directions and can therefore maximally couple into each l-mode, with the electric fields orientating such that the maxima aligns with the location of the QE. The electric field that scatters from a 1μm diameter isolated gold NP, due to an x-polarised QE in close proximity, is decomposed into its modal contributions and is shown in Figure S5.

In comparison, a plane wave induces successive $\pi/2$ rotations between consecutive l-modes, which leads to alternating maxima and minima in the excitation experience by a QE placed close to the NP—as we have seen in the main manuscript (Figure 3). For a QE sat just above the NP along the x-axis, as in the analytical model considered here, only odd l-modes will be efficiently excited. For this polarisation, measuring along the y-axis reverses the nodes and antinodes, revealing primary coupling into the even l-modes. However, the maxima along the y-axis lie away from the NP surface—where the greatest enhancements occur in nanocavities—and so cannot efficiently couple into even l-modes in nanoantenna systems.

For a particular far-field plane wave excitation we have seen how this restricts the value of m, and have shown in Figure 3 the $|E_x|$ decomposition for an x-polarised plane wave. In Figure S6 and S7 we respectively show the corresponding $|E_y|$ and $|E_z|$ components, which reinforce how the coupling is limited to odd-l modes only.

In addition to demonstrating the selective nature of plane wave excitation, Figure S8 extends on the radial dependence analysis to decompose how three different sized NPs couple into the first three l-modes, respectively. With radii of 30nm, 100nm and 500nm, the plane wave can efficiently couple into the $l = 1$ mode of each NP. In addition to the selective nature of the plane wave, for the $l = 2$ mode we also observe the quasi-static effects emerging for the smallest NP—where the NP is so small compared to the incoming field that it barely experiences this mode. Similarly with the $l = 3$ mode, due to phase propagation effects even

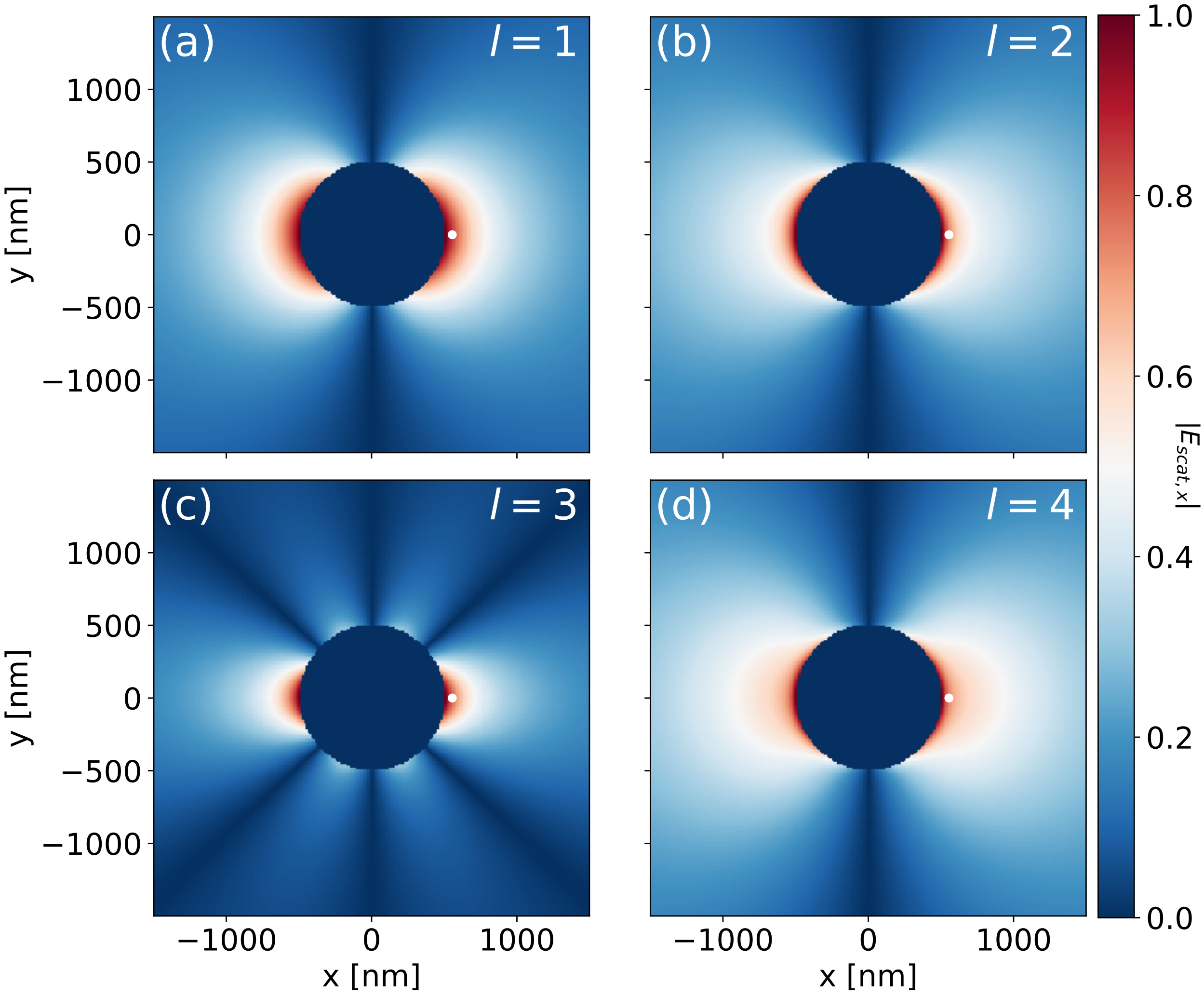

Figure S5: An analytical modal decomposition of the electric fields around an isolated gold NP, due to an x-polarised QE placed 0.5nm above the NP in the x-axis. The white dot indicates the position of the QE. (a) $l = 1$. (b) $l = 2$. (c) $l = 3$. (d) $l = 4$.

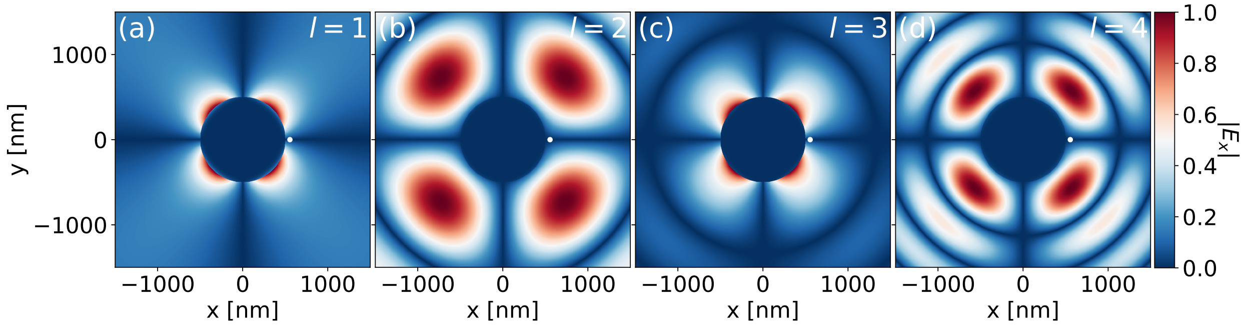

Figure S6: Total $|E_y|$ fields of a 1μm NP excited by an x-polarised plane wave, obtained from the multipolar decomposition for modes (a) $l = 1$, (b) $l = 2$, (c) $l = 3$, (d) $l = 4$. The white dot indicates the position where the γ_{exc} is measured.

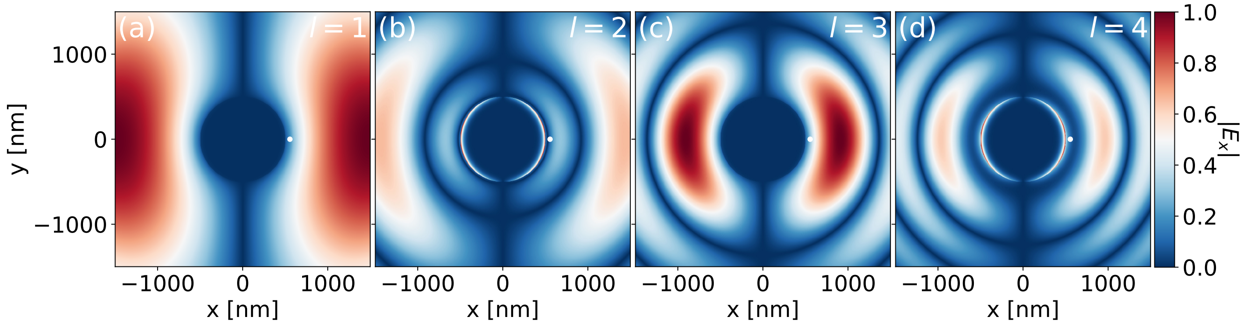

Figure S7: Total $|E_z|$ fields of a 1μm NP excited by an x-polarised plane wave, obtained from the multipolar decomposition for modes (a) $l = 1$, (b) $l = 2$, (c) $l = 3$, (d) $l = 4$. The white dot indicates the position where the γ_{exc} is measured.

the 100nm radius NP is unable to efficiently in-couple.

In comparison, Figure S9 demonstrates how these same three NPs are able to emit energy due to a coupling with a close QE. For the $l = 1$ mode, all three NPs are able to efficiently scatter the energy that the QE couples into them. The QE is again able to couple energy into the $l = 2$ mode of each of the NPs, however, the larger NPs are able to scatter this energy away much more efficiently—hence the more extended exponential decay of the fields from the NP's surface. This is similarly true for the $l = 3$ mode.

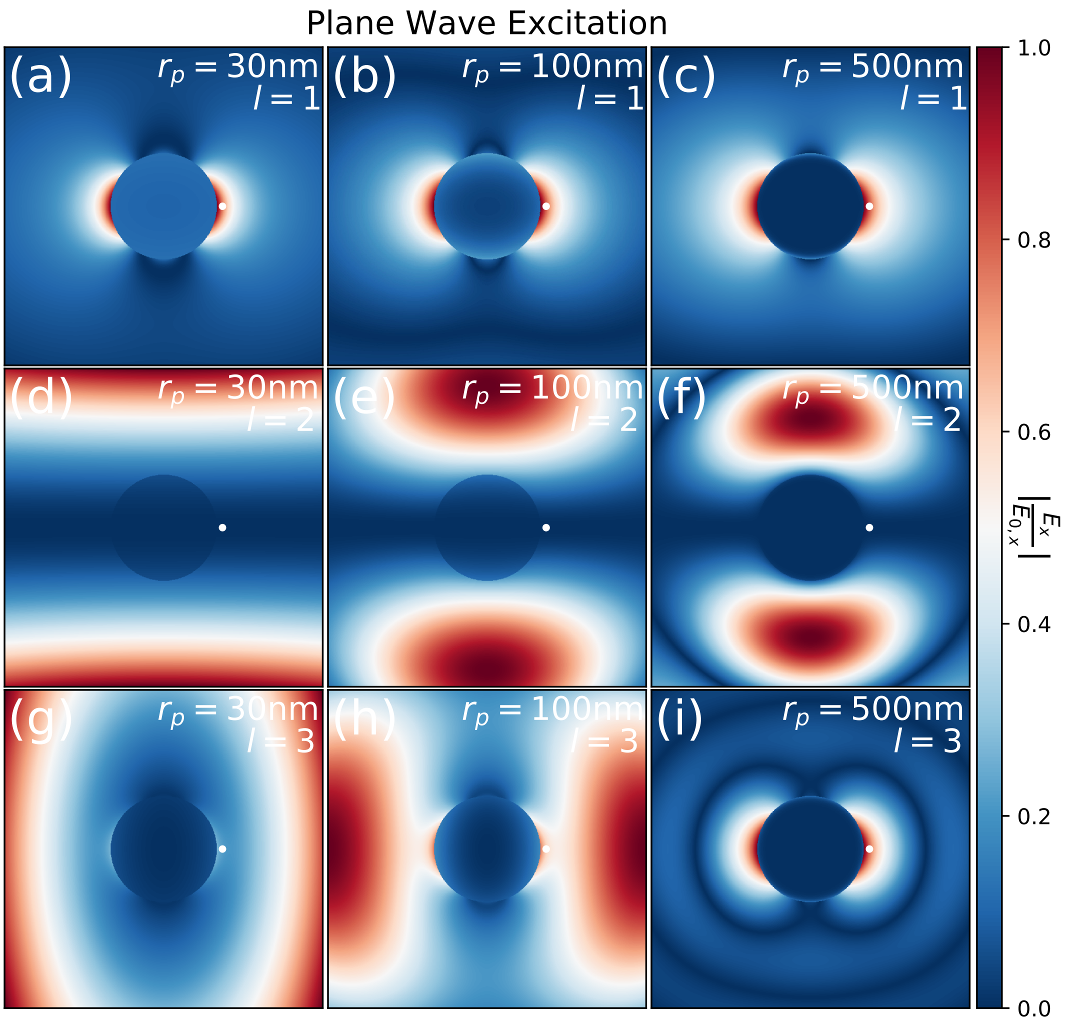

Figure S8: The modal contribution to the electric field around an isolated gold NP due to a plane wave excitation. (a-c) $l = 1$ mode, (d-f) $l = 2$ mode, and (g-i) $l = 3$ mode. (a,d,g) $r_p = 30$nm, (b,e,h) $r_p = 100$nm, and (c,f,i) $r_p = 500$nm. The white dot indicates the location where the fields are measured when calculating the γ_{exc}. For clarity, each image is scaled to size of the NP.

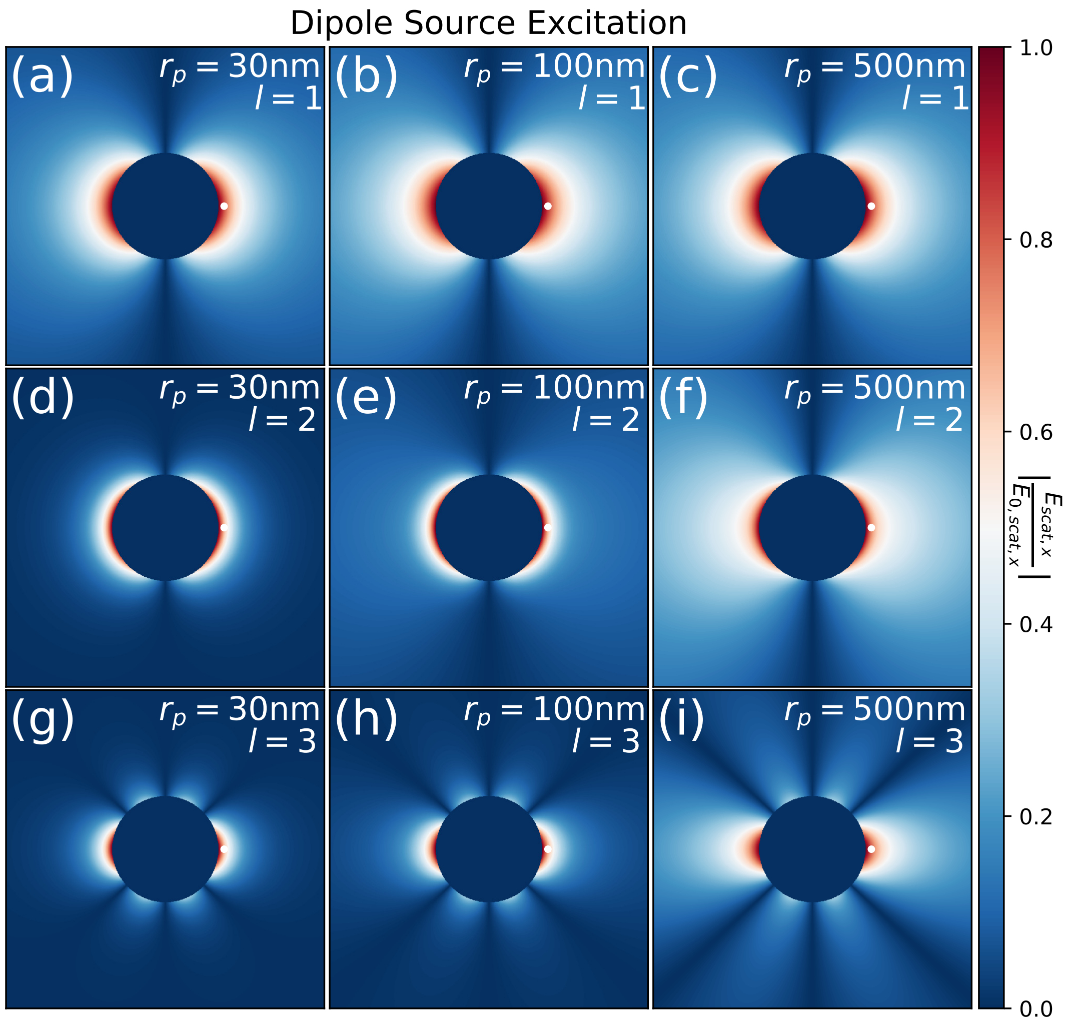

Figure S9: The modal contribution to the electric field around an isolated gold NP due to a dipole excitation. (a-c) $l = 1$ mode, (d-f) $l = 2$ mode, and (g-i) $l = 3$ mode. (a,d,g) $r_p = 30$nm, (b,e,h) $r_p = 100$nm, and (c,f,i) $r_p = 500$nm. The white dot indicates the position of the QE. For clarity, each image is scaled to size of the NP.

S8 Normal and In-Plane Excitations

To demonstrate the different set of modes that the normal plane wave excitation couples into, Figure S10 compares the in-plane and normal excitations, relative to the mirror. Due the red-shifting of this nanocavity, the modes of each excitation incidence are spectrally separated and do not interfere.

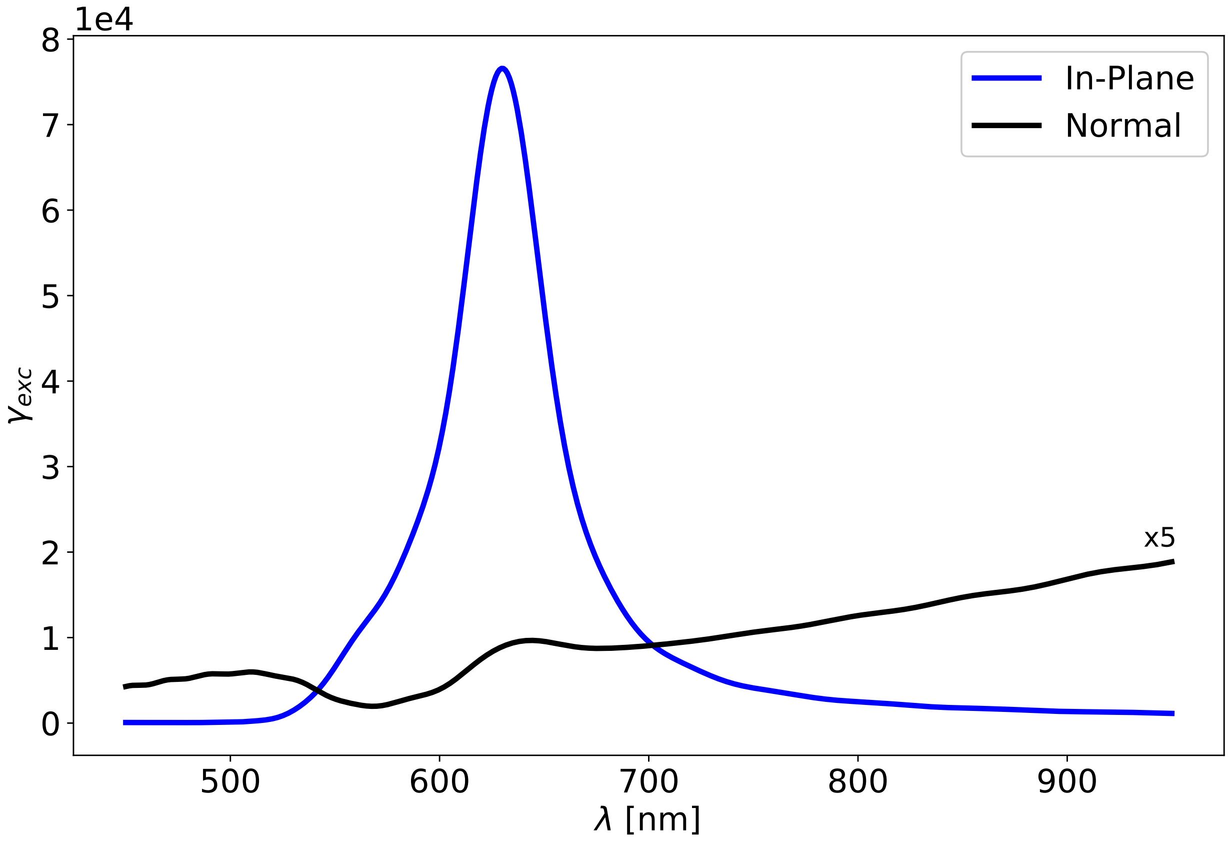

Figure S10: Numerical comparison of the γ_{exc} of the NPoM geometry for different plane wave incidences relative to the mirror: in-plane (blue) and normal (black). Here the NPoM consists of a 60nm NP assembled $d = 1$nm above an infinite, flat substrate.

S9 Nanoantenna Emitter Positioning

To explore the effect of the dipole source positioning within the cavity of nanoantenna systems, Figure S11 and S12 compares γ_{exc} and γ_{rad} at five different emitter positions along the x-axis for the symmetric dimer antenna and NPoM geometries, respectively. In both cases, the γ_{exc} and γ_{rad} reduce with distance away from the centre of the cavity due to the lower confinement and field enhancement. Due to the singular $l = 1$ mode present in the hybridisation of the dimer cavity, γ_{exc} and γ_{rad} remain equal at positions away from the cavity centre. Although the NPoM system exhibits unequal coupling rates, it retains the same relative amplitude between γ_{exc} and γ_{rad} with positions away from the cavity centre. Note that the resonant frequency and relative line width (fraction of non-radiative decay) remain constant.

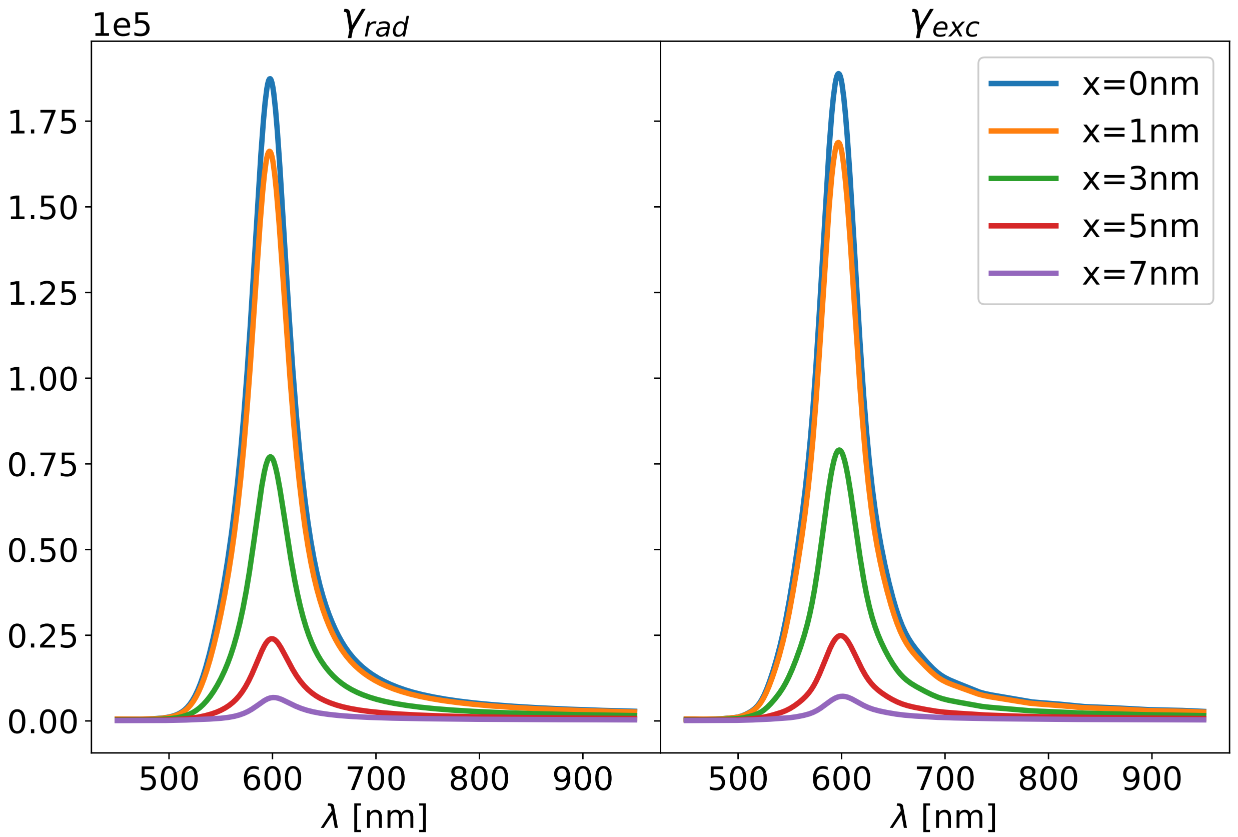

Figure S11: Numerical comparison of the γ_{rad} and γ_{exc} of the symmetric quasi-static dimer antenna of $2r_{p,1} = 2r_{p,1} = 60$nm, for a series of emitter positions along the x-axis: $x = 0$nm to $x = 7$nm.

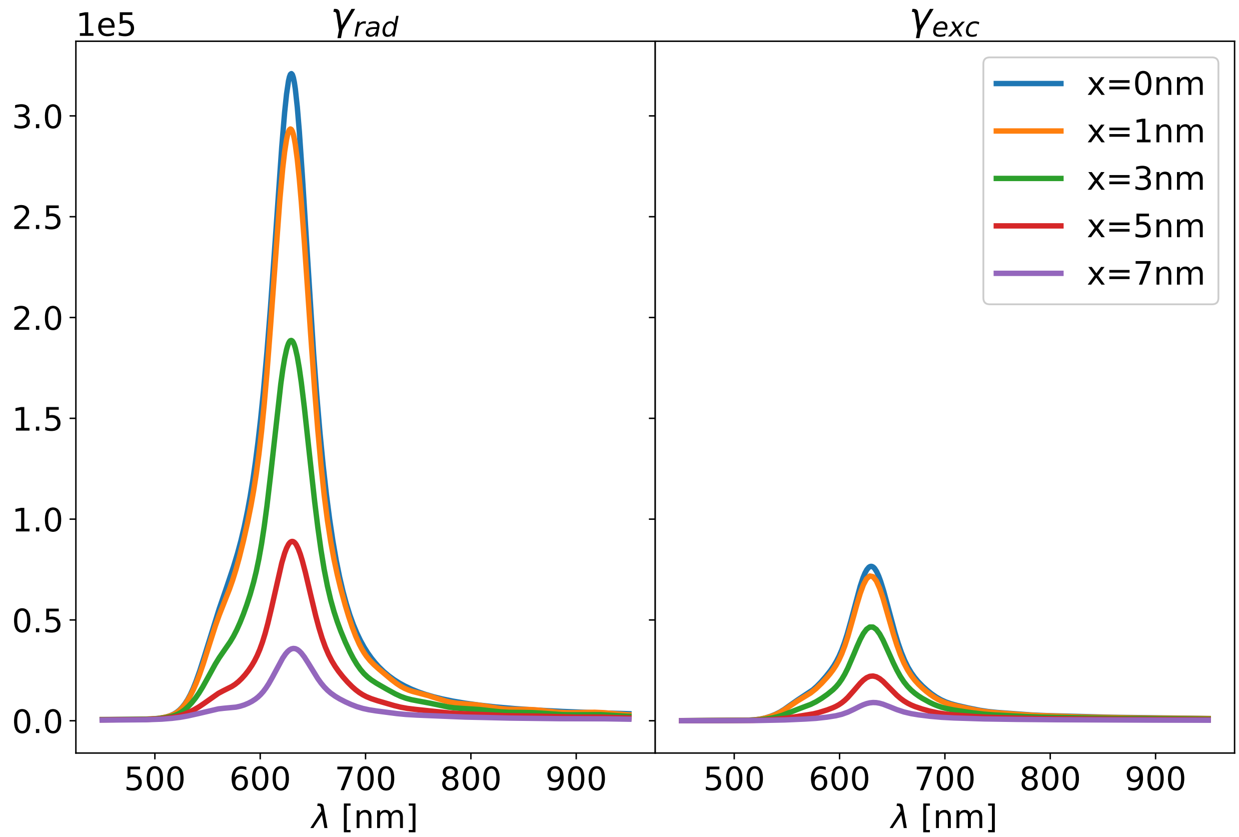

Figure S12: Numerical comparison of the γ_{rad} and γ_{exc} of the NPoM geometry of a $2r_p =$ 60nm gold NP assembled $d = 1$nm above a gold substrate, for a series of emitter positions along the x-axis: $x = 0$nm to $x = 7$nm.

S10 Asymmetric Dimer Coupling

For a large dimer system consisting of a $r_{p,1} = 30$nm NP sat 1nm above a $r_{p,2} = 500$nm NP, we observe a corresponding coupling profile as shown in Figure S13. This exhibits a very similar coupling dynamics as that seen for the NPoM system, which experiences: a red-shifting of the resonance, an enhancement of both the γ_{exc} and γ_{rad}, and an unequal in- and out-coupling of energy.

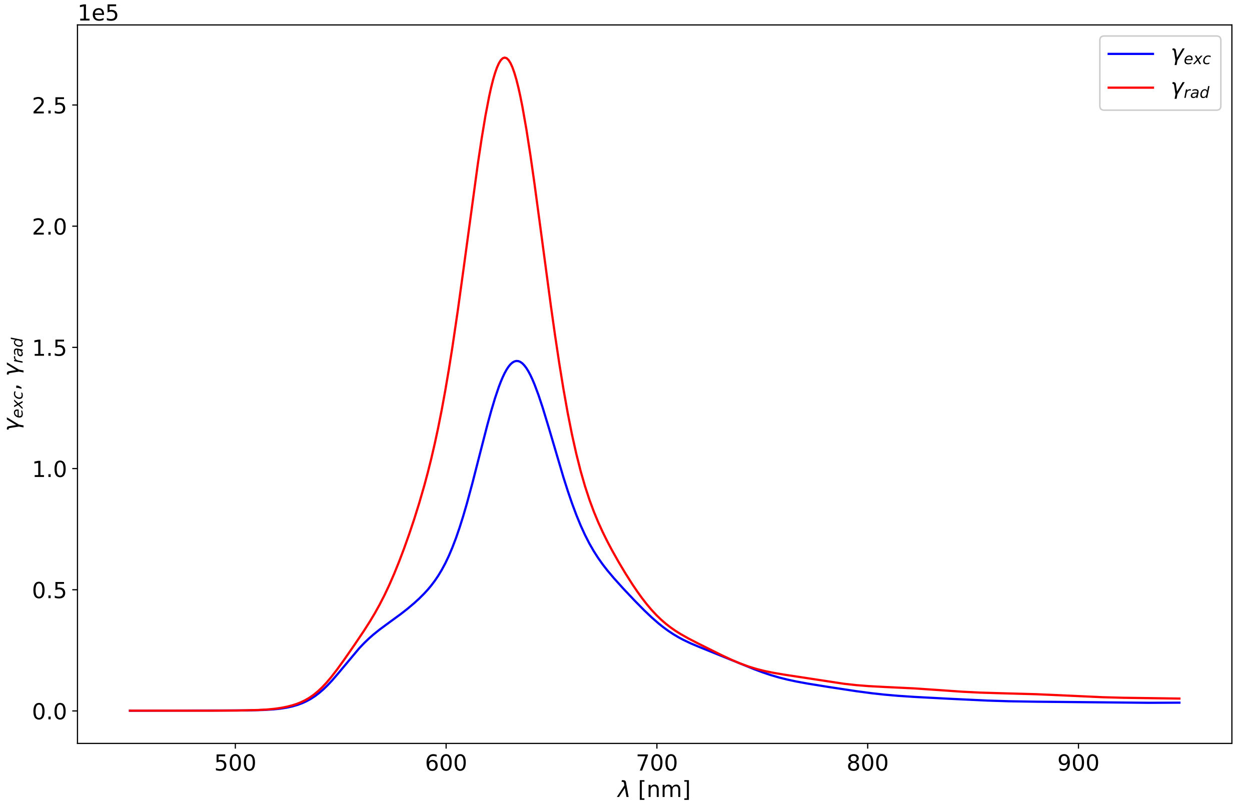

Figure S13: The γ_{exc} (blue) and γ_{rad} (red) obtained from FDTD calculations for an asymmetric dimer system consisting of a 60nm diameter gold NP sat 1nm above a 1μm diameter gold NP. The position of the dipole source when determining the γ_{rad}, and the location where the fields are measured when calculating the γ_{exc}, are both at the centre cavity.

S11 Nanoantenna Formation

To develop on the separation dependence of Figure 5 in the main manuscript, here we comment further on how specific in- and out-coupling ratios are obtained by comparing the the coupling rates of the isolated components with the full nanoantenna. In Figure S14 we compare the coupling profiles of the $2r_p = 60$nm and $2r_p = 500$nm NPs with the $d = 1$nm $2r_{p,1} = 60$nm—$2r_{p,2} = 500$nm dimer system. The yellow region highlights the primary overlap and enhancement of the large NPs $l = 3$ mode with the small NPs singular $l = 1$ mode—once the red-shift from the hybridisation is taken into account. As this falls directly on reasonably well separated odd-l modes, the response of the dimer has near perfect equal γ_{exc} and γ_{rad}. Small differences in the tail on the right of the peak occur due to the tail of the small NPs $l = 1$ mode and the relative closeness of the large NPs $l = 2$ mode.

Similarly, Figure S15 shows the same information when the lower NP is increased to a radius of $r_p = 500$nm. As the modes of the isolated NPs now primarily overlap with an even ($l = 6$) mode—after the hybridisation red-shift—the overall response of the antenna heavy reflects these differences: with the γ_{rad} much larger than the γ_{exc}.

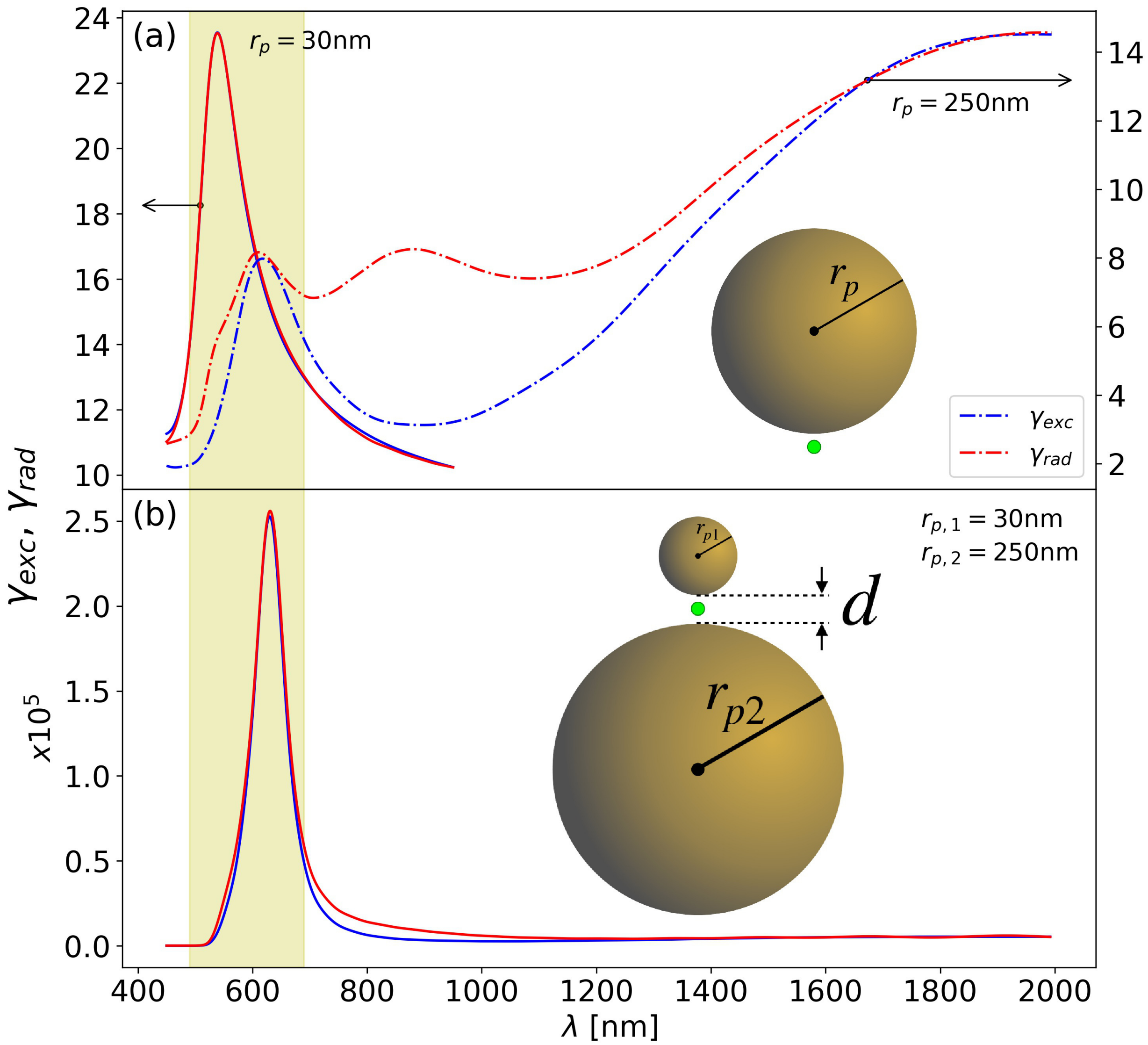

Figure S14: Numerical comparison of the γ_{exc} (blue) and γ_{rad} (red) as a function of wavelength for (a) a $2r_p = 60$nm isolated gold NP (left, full) and $2r_p = 500$nm isolated gold NP (right, dashed), and (b) an asymmetric gold dimer antenna consisting of a $2r_{p,1} = 60$nm NP positioned $d = 1$nm above a $2r_{p,2} = 500$nm NP. The green dot indicates the position of the QE when determining the γ_{rad}, and the location where the fields are measured when calculating the γ_{exc}.

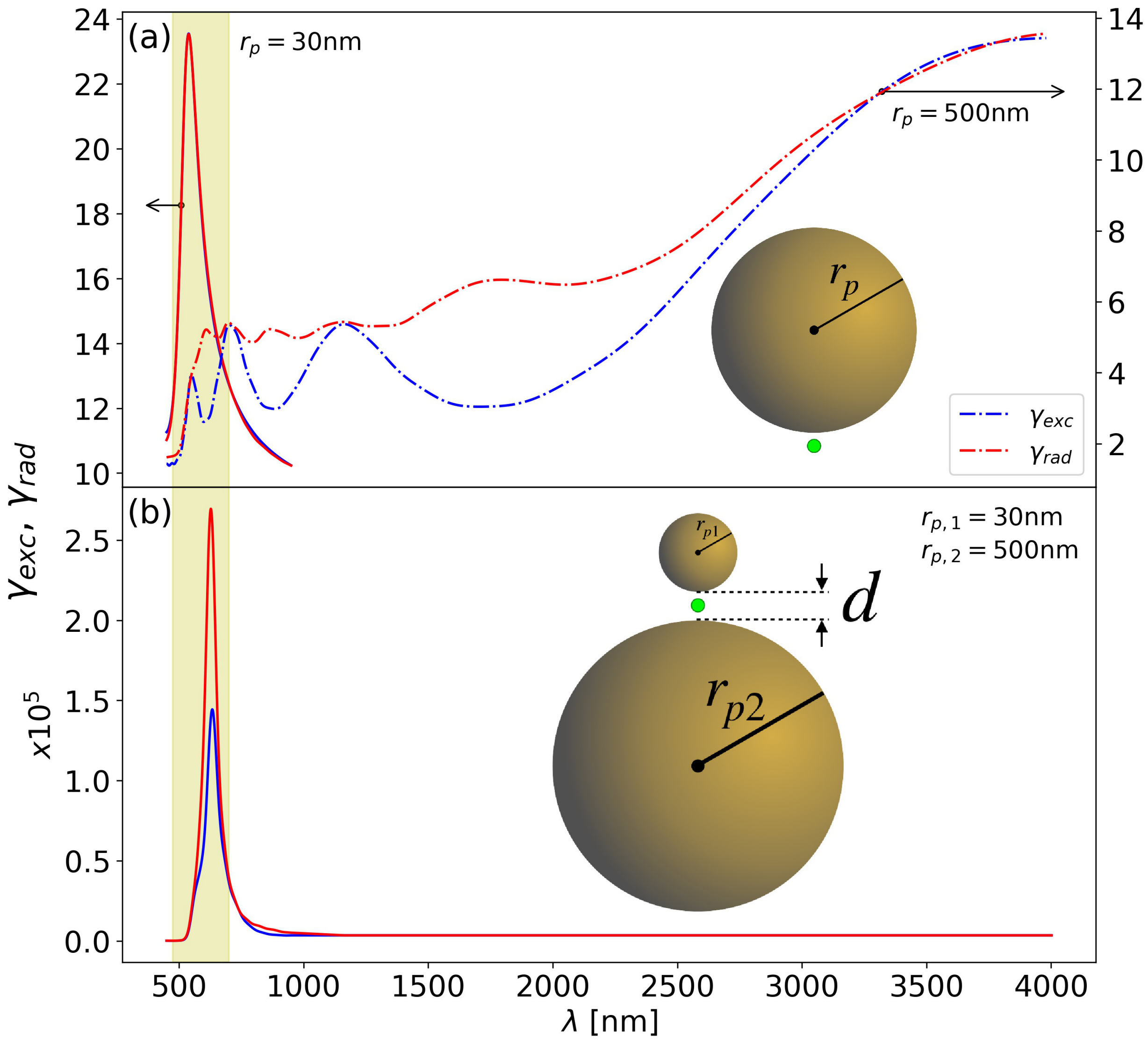

Figure S15: Numerical comparison of the γ_{exc} (blue) and γ_{rad} (red) as a function of wavelength for (a) a $2r_p = 60$nm isolated gold NP (left, full) and $2r_p = 1\mu$m isolated gold NP (right, dashed), and (b) an asymmetric gold dimer antenna consisting of a $2r_{p,1} = 60$nm NP positioned $d = 1$nm above a $2r_{p,2} = 1\mu$m NP. The green dot indicates the position of the QE when determining the γ_{rad}, and the location where the fields are measured when calculating the γ_{exc}.

References

(1) Bohren, C. F. *Absorption and scattering of light by small particles*; 1983.

(2) Frezza, F.; Mangini, F.; Tedeschi, N. Introduction to electromagnetic scattering: tutorial. *Journal of the Optical Society of America A* **2018**, *35*, 163–173.

(3) Lumerical Inc. https://www.lumerical.com/.

(4) COMSOL Multiphysics®v. 5.0. www.comsol.com. COMSOL AB, Stockholm, Sweden.
